# Effect of arbuscular mycorrhizal fungi on the responses of *Ageratina adenophora* to *Aphis gossypii* herbivory

**DOI:** 10.3389/fpls.2022.1015947

**Published:** 2022-10-17

**Authors:** Ewei Du, Yaping Chen, Yahong Li, Fengjuan Zhang, Zhongxiang Sun, Ruoshi Hao, Furong Gui

**Affiliations:** ^1^ State Key Laboratory for Conservation and Utilization of Bioresources in Yunnan, College of Plant Protection, Yunnan Agricultural University, Kunming, China; ^2^ Department of Plant Quarantine, Yunnan Plant Protection and Quarantine Station, Kunming, China; ^3^ College of Life Science, Hebei University, Baoding, China; ^4^ Department of Industrial Development, Yunnan Plateau Charateristic Agriculture Industry Research Institute, Kunming, China

**Keywords:** *Ageratina adenophora*, arbuscular mycorrhizal fungi, *Aphis gossypii*, tolerance, resistance

## Abstract

The invasive weed *Ageratina adenophora* can form a positive symbiotic relationship with native arbuscular mycorrhizal fungi (AMF) to promote its invasion ability. However, the function of AMF during the feeding of *Aphis gossypii* in *A. adenophora* was poorly understand. This study aimed to investigate the effects of two dominant AMF (*Claroideoglomus etunicatum and Septoglomus constrictum*) on *A. adenophora* in response to the feeding of the generalist herbivore *A. gossypii*. The results showed that *A. gossypii* infestation could significantly reduce the biomass, nutrient and proline contents *of A. adenophora*, and increase the antioxidant enzyme activities, defense hormone and secondary metabolite contents of the weed. Compared with the *A. gossypii* infested *A. adenophora*, inoculation *C*. *etunicatum and S*. *constrictum* could significantly promote the growth ability and enhanced the resistance of *A. adenophora* to *A. gossypii* infestation, and the aboveground biomass of *A. adenophora* increased by 317.21% and 114.73%, the root biomass increased by 347.33% and 120.58%, the polyphenol oxidase activity heightened by 57.85% and 12.62%, the jasmonic acid content raised by 13.49% and 4.92%, the flavonoid content increased by 27.29% and 11.92%, respectively. The survival rate of *A. gossypii* and density of nymphs were significantly inhibited by AMF inoculation, and the effect of *C. etunicatum* was significantly greater than that of *S. constrictum*. This study provides clarified evidence that AMF in the rhizosphere of *A. adenophora* are effective in the development of tolerance and chemical defense under the feeding pressure of insect herbivory, and offer references for the management of the *A. adenophora* from the perspective of soil microorganisms.

## 1 Introduction

*Ageratina adenophora* (Sprengel) originated in Central America and is regarded as one of the most serious invasive species in Asia, Africa, and Oceania (Poudel et al., 2019; Tang et al., 2019). It was introduced into Yunnan Province in China, from Myanmar in the 1940s and is now distributed in southwestern and central China including Yunnan, Guizhou, Sichuan, Guangxi, Tibet, and Chongqing (Wang and Wang, 2006; Gui et al., 2009). The weed may be expanding its distribution due to adaptation to local climates in its introduced ranges (Datta et al., 2019) or its stress tolerance and phenotypic plasticity, allowing it to outcompete native plants (Poudel et al., 2020). Most importantly, this weed induces serious ecological damage by establishing monocultures in places where native plants once flourished (Gao et al., 2013). It has caused huge economic losses to agriculture, forestry, and animal husbandry (Ren et al., 2021). It is estimated to cause economic losses to animal husbandry and grassland ecosystem services of RMB 0.99 and 2.63 billion per year, respectively (Wang et al., 2017a), and effective control of this species is urgently needed (Song et al., 2017).

With the invasion and expansion of *A. adenophora*, native polyphagous insects gradually established ecological relationships with the weed (Heystek et al., 2011). A polyphagous insect, cotton aphids (*Aphis gossypii* Glover, Homoptera, Aphididae) was found to have colonized *A. adenophora* in our field investigation. Aphids can reproduce rapidly within a few days, hiding on the lower surface of the leaves and the core of the young leaves of *A. adenophora*, feeding on the phloem sap of *A. adenophora* and causing a high degree of damage to the leaf cells (Cheng et al., 2007). The honeydew excreted by aphids during feeding can decrease plant photosynthesis and lead to mold parasitism, which affects plant growth (Kuśnierczyk et al., 2008). Previous study has shown that *A. adenophora* damage caused by *A. gossypii* increases significantly with increasing aphid density and feeding time, which can reduce the growth of *A. adenophora*, and thus inhibit the expansion of *A. adenophora* to some extent (Lin et al., 2020).

Arbuscular mycorrhizal fungi (AMF) are essential ecological components of soil communities and form obligate mutualistic associations with 80% of terrestrial vascular plants (Smith, 2008; Brundrett and Tedersoo, 2018). It has been widely recognized that AMF can enhance host plant defense against pathogens and insect herbivory (Cameron et al., 2013; Martinez-Medina et al., 2016). The AM fungal-mediated mechanisms that increase plant resistance against herbivores and pathogens are through the alteration of both plant tolerance and chemical defense (Rivero et al., 2021; Frew et al., 2022). Exotic plant can selectively accumulate AM fungal communities in their rhizosphere that are different from those of native plants, thus affecting their invasion (Zhang et al., 2017; Zhang et al., 2018). *A. adenophora* invasion was reported to cause changes in the AM fungal diversity of native plants, *Septoglomus constrictum* was one of the dominant AMF on the roots and in the rhizosphere soil of *A. adenophora* (Xiao et al., 2014). Our previous study on AMF in the rhizosphere soil of *A. adenophora* found that *S. constrictum* and *Claroideoglomus etunicatum* were the most abundant species. The dominant AMF in the rhizosphere of invasive plants, such as *Flaveria bidentis* (Chen et al., 2021), *Solidago canadensis* (Qi et al., 2022), *Ambrosia artemisiifolia* (Zhang et al., 2018), can induce positive feedback effects on invasive plant. Several studies showed that the dominant AMF in the rhizosphere of *A. adenophora* can improve photosynthesis, increase nutrient content, and enhance its competitive advantage over native plants (Li et al., 2016; Tan et al., 2021). However, it was not clear whether the dominant AMF altered by the invasion of *A. adenophora* could enhance the resistance of *A. adenophora* to herbivorous insects. Therefore, this study aimed to investigate the effects of different dominant AMF on the invasive plant *A. adenophora* in response to the feeding of the generalist herbivore *Aphis gossypii* and to provide a reference for the management of the *A. adenophora*.

## 2 Materials and methods

### 2.1 Plants and soil preparation

The soil and seeds of *A. adenophora* were obtained from the field near the campus of Yunnan Agricultural University (25°08′30″N, 102°45′13″E, with an elevation of 1,940 m). The seeds were sown in potting soil in seedling trays and placed in artificial climate chambers with parameters set as follows: 25°C, 70% relative humidity, a photoperiod of 16 h light/8 h dark, and an illumination intensity of 20,000 LX supplied by 40 W fluorescent lamps (OPPLE, Deyuansicheng Biotech Company, Beijing, China). The soil was ground fine enough to pass through a 2 mm sieve and was mixed with vermiculite (v/v = 1:1) [(Mg,Fe,Al)_3_[(Si,Al)_4_O_10_ (OH)_2_]. 4H_2_O] that was purchased from Dounan Plant and Flower Co., Ltd., Kunming, China. The basic properties of the soil were as follows: the pH (w/v water = 1:5) was 6.25, the organic matter content was 15.502 g/kg, total nitrogen was 0.899 g/kg, total phosphorus was 0.351 g/kg, total potassium was 40.03 g/kg, available nitrogen was 20.28 µg/g, available phosphorus was 5.089 µg/g, and available potassium was 32.32 mg/kg. The mixtures were autoclaved at 121°C for 2 h.

### 2.2 AM fungal preparation and insect rearing

The AMF used in the study were mycorrhizal inoculum *S. constrictum* and *C. etunicatum*, which were the dominant AMF in the rhizosphere soil of *A. adenophora* (Daniels and Skipper, 1982; Morton, 1990). After expanding the culture with maize as the host plant, the inoculum was a mixture of spores and mycelia. The spore density was defined as the number of AM fungal spores and sporocarps in 100 g of soil. Based on the spore density in the rhizosphere soil of *A. adenophora* in the field, 20 spores/100 g soil were selected for the experiment.

Cotton aphids, *A. gossypii*, were collected from *A. adenophora* plants in the suburb of Kunming and reared on *A. adenophora* in the greenhouse for ten generations to make sure the cotton aphids used in the experiment were from the same population.

### 2.3 Experimental design

The pot experiment was carried out in the greenhouse at Yunnan Agricultural University. Three treatments were used to determine the effects of different AMF on *A. adenophora* in response to the feeding by *A. gossypii*: (1) C, uninoculated treatment; (2) SC, inoculation with *S. constrictum*; (3) CE, inoculation with *C. etunicatum*. Each treatment was set with two levels: with *A. gossypii* infestation and without *A. gossypii* infestation. Before planting, a total of 40 g of inoculum (20 g spores/100 g soil) was mixed with 1 kg of the growth substrate. The control treatment was inoculated with the same amount of sterile inoculum. One seedling was transplanted into a plastic pot (17.8 cm diameter and 10.4 cm height) at 5 cm and cultivated in the same artificial climate chamber. All pots were watered every two days and re-randomized twice per week to minimize the position effect. Each pot was placed individually in a 100 mesh cage (45 cm length, 30 cm width). After the plants had grown for 2 months, 60 1st-instar aphid nymphs were released on the top of two tender leaves of each plant in the cages (Lin et al., 2020). Until all the aphid inoculated died, the infested leaves were picked to measure the contents of plant nutrients, stress resistance substances, secondary metabolites, defense hormone contents, and antioxidant enzyme activities. The experiment has five replicates for each treatment.

### 2.4 Measurements

#### 2.4.1 Plant aboveground and belowground biomass

The aboveground and belowground parts of *A. adenophora* were harvested separately. The first three pairs of leaves (six leaves, about 1-1.2g fresh weight) from the top of each treatment of five plants were removed and combined for physiological measurements. The belowground parts were washed free from the soil, and a quantitative part (0.2g) of the roots was taken from each treatment to determine the mycorrhizal colonization rate. After measuring the root growth characteristics, the aboveground and belowground parts of *A. adenophora* were oven-dried at 80°C for 72 h, and the aboveground and root biomass were measured respectively.

#### 2.4.2 Root growth characteristics

After being carefully washed, the roots were scanned using a root scanner (Epson Expression 10000XL; Epson, Long Beach, CA, USA). The root growth characteristics (root length, root surface area, root diameter and root volume) were determined using WinRhizo Software (Regent Instruments Inc., Québec City, QC, Canada).

#### 2.4.3 Nutrients contents, malondialdehyde and free proline content

Nutrients (soluble sugar, protein, and starch) were quantified in potassium phosphate buffer (KPB) (50 mM, pH= 7.5) using extracts of fresh leaves (0.1 g). These extracts were filtered through four cheese cloth layers and centrifuged at 15,500 rpm for 15 min at 4°C. The supernatant was collected and stored at 4°C for soluble protein and sugar determinations. The contents of soluble sugar and protein were measured following the instructions of the kits (A045-4-2 for soluble protein, A145-1-1 for soluble sugar; Nanjing Jiancheng Bioengineering Institute, Nanjing, China). The pellet was homogenized in 2 mL distilled water and mixed with 2mL perchloric acid (9.2 mol/L) (Jarvis and Walker, 1993). After centrifugation at 4000 rpm for 10 min, the 2 mL supernatant was mixed with 5mL anthrone-sulfuric acid reagent and incubated for 10 min at 100°C. Their absorbance at 620 nm was measured using a microplate reader (Varioskan LUX, Thermofisher, USA), the soluble starch content was calculated. The fresh leaves (0.1g) were homogenized in 4mL 10% trichloroacetic acid (w/v) at 4°C and were centrifuged at 4000 rpm for10 min. The supernatant was used to measure leaf malondialdehyde (MDA) content by kit (A003-1-2, Nanjing Jiancheng Bioengineering Institute, Nanjing, China). For the leaf proline, the 0.1g fresh leaves were weighed to extract proline using 10mL solution of 3% sulfosalicylic acid and then centrifuged at 10,000g for 5 min. The supernatant was used to measure proline content by kit (A107-1-1, Nanjing Jiancheng Bioengineering Institute, Nanjing, China). The total chlorophyll was estimated using the spectrophotometric method (Lichtenthaler, 1987) and was calculated as mg/g fresh weight.

#### 2.4.4 Antioxidant enzyme activities, defense hormones, and secondary metabolite contents

About 0.2g of the leaf samples were homogenized in 1.8mL of 0.1 M phosphate buffer (pH 7.0) on ice, followed by centrifugation at 10,000rpm for 10 min. The supernatant was collected to analyze antioxidant enzymatic activities. Superoxide dismutase (SOD), polyphenol oxidase (POD), catalase (CAT), and polyphenol oxidase (PPO) of leaf tissues were analyzed using the detection kits (A001-1-2 for SOD, A084-3-1 for POD, A007-1-1 for CAT, and A136-1-1 for PPO; Nanjing Jiangcheng Bioengineering Institute, Nanjing, China) according to the manufacturer’s instruction. Weigh 0.2g of young leaves, add 1 mL of methanol to fully grind, and add 9mL phosphate buffer to homogenize. After centrifugation at 4000 rpm for 10 min, JA and SA were determined using ELISA kits (Jiangsu Boshen Biotechnology Co., Ltd., Nanjing, China), following the manufacturer’s protocol. Weigh 0.1g of fresh leaves in 2 mL of 60% ethanol solution and homogenize at 4°C. Extraction was performed at 60°C for 1 h and centrifuged at 10,000rpm for 10 min. The total phenolic and flavonoid content of plants was detected using a kit (A143-1-1 for total phenols, A142-1-1 for flavonoids; Nanjing Jiangcheng Bioengineering Institute, Nanjing, China). The tannic acid content was measured referring to Pang et al. (2006) and Lin et al. (2020). The optical density (OD) values of each reaction mixture were measured by a microplate reader (Varioskan LUX, Thermofisher, USA).

#### 2.4.5 Mycorrhizal colonization rate

Root samples of *A. adenophora* were collected about 3 months after the plants were inoculated with AMF. The percentage colonization of AMF in roots of *A. adenophora* was observed using the magnified intersection method (Biermann and Linderman, 1981; Zhang et al., 2018). Specifically, the roots were cut into 1–2 cm segments, cleared with 10% KOH at 90°C for 15 min and acidified with 2% HCl at 30°C for 10 min. The treated root materials were stained with 0.1% acid fuchsin solution (0.1 g acid fuchsin dissolved in 63 ml sterile dH_2_O, with the addition of 63 ml glycerin, and 875 ml lactic acid stored at room temperature) at 90°C for 30 min. A total of 200 root segments were measured for each replicate, and the percentage colonization of each segment was determined by the colonization in that region if any hyphae, vesicles, or arbuscules were visible.

#### 2.4.6 Aphid survival rate and fecundity

After the *A. gossypii* infestation, the number of *A. gossypii* was observed and recorded once a day, and the aphid survival rate was calculated.

Aphid survival rate = number of aphids recorded per day/number of aphids initially inoculated

When the nymphs grew to the adult aphid and began to reproduce, the number of nymphs produced by aphids was observed and recorded every day, and the 1st-instar aphid nymphs were removed in time until all the aphid inoculated died.

### 2.5 Statistical analysis

Before analyses, all data were tested for normality using the Shapiro–Wilk test. All data met the normality assumption. Data are mean ± SE of five replicates in each treatment. Differences in the effect of different insect feeding (with or without *A. gossypii* infestation) and AM fungal treatments (C, SC, CE) on the activity in protective enzymes, defensive substances, hormones, and secondary metabolite contents in *A. adenophora* were analyzed using two-way analysis of variance (ANOVA). Different in the effect of different AM fungal treatments on the rate of surviving *A. gossypii* and nymphal production were analyzed using a one-way analysis of variance. Differences between the two groups were compared using independent samples *t*-tests. Significant differences between treatments were based on the LSD test. Statistical significance was set at *P* < 0.05. All analyses were conducted using SPSS 21.0 (SPSS Inc., Chicago, IL, United States).

## 3 Results

### 3.1 Effects of inoculation with AMF and *A. gossypii* herbivory on the aboveground and belowground biomass of *A. adenophora*

**Figure 1:**
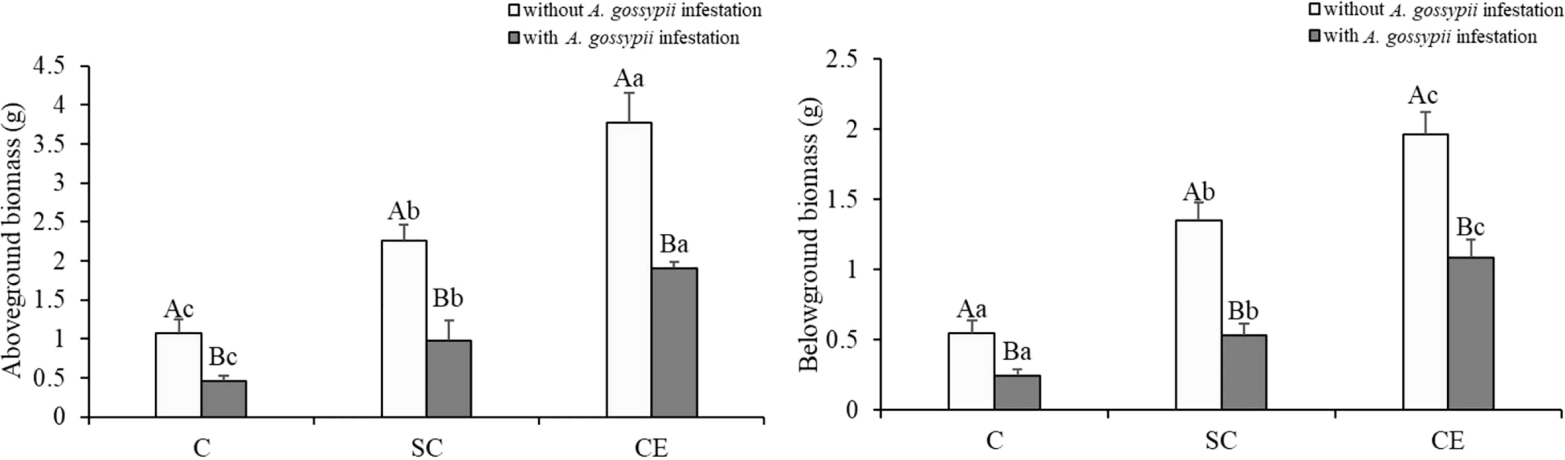
Effect of inoculation with AMF and *A. gossypii* herbivory on the aboveground and root biomass of *A. adenophora*. The three treatments were (1) C, inoculation with sterilized AMF, (2) SC, inoculation with *S. constrictum*, (3) CE, inoculation with *C. etunicatum*. Different lowercase letters above the bars indicate significant differences among the three inoculation treatments (Duncan’s test, *P* < 0.05). Different uppercase letters above the bars indicate significant differences between treatments with or without *A. gossypii* infestation (*t*-test, *P* < 0.05) Error bars represent ± 1SE of the mean (n = 5).

Inoculation of two kinds of AMF (*S. constrictum* and *C. etunicatum*) increased the aboveground and belowground biomass of *A. adenophora* (aboveground: SC: *F*_(2,24)_ = 75.750, *P* < 0.001, CE: *F*_(2,24)_ = 446.210, *P* < 0.001; belowground: SC: *F*_(2,24)_ = 119.971, *P* < 0.001, CE: *F*_(2,24)_ = 508.661, *P* < 0.001, **Table 1**). Feeding of *A. gossypii* significantly reduced the biomass of *A. adenophora* (aboveground: *F*_(2,24)_ = 278.644, *P* < 0.001; belowground: *F*_(2,24)_ = 302.028, *P* < 0.001, **Table 1**), the aboveground and root biomass of *A. gossypii* infestation decreased by 57.70% and 55.74% compared with the uninoculated treatment (**Figure 1**). However, the symbiotic effect of AMF could significantly increase the growth of *A. adenophora* in *A. gossypii* infestation (*P* < 0.001, **Figure 1**). The aboveground biomass of *A. adenophora* inoculated with *S. constrictum* (SC) and *C. etunicatum* (CE) increased by 114.73% and 317.21%, and the root biomass increased by 120.58% and 347.33% compared with the uninoculated treatment. The effect of CE treatment on the aboveground biomass of *A. adenophora* with infestation *A. gossypii* was greater than that of SC treatment (SC*A. gossypii: *F*_(2,24)_ = 11.580, *P* = 0.002, CE*A. gossypii: *F*_(2,24)_ = 40.776, *P* < 0.001, **Table 1**).

**Table 1:** Two-way ANOVAs of the effects of inoculation with AMF and *Aphis gossypii* herbivory on the growth indicators of *A. adenophora*.

Parameters	*S. constrictum* treatment		*C. etunicatum* treatment		*A. gossypii* treatment		S. constrictum*A. gossypii treatment		C. etunicatum*A. gossypii treatment	
	*F*	*P*	*F*	*P*	*F*	*P*	*F*	*P*	*F*	*P*
Aboveground biomass	75.750	<0.001***	446.210	<0.001***	278.644	<0.001***	11.508	0.002**	40.776	<0.001***
Root biomass	119.971	<0.001***	508.661	<0.001***	302.028	<0.001***	25.956	<0.001***	32.274	<0.001***
Soluble sugar	35.862	<0.001***	329.338	<0.001***	135.764	<0.001***	0.014	0.907	7.893	0.010*
Soluble protein	135.392	<0.001***	373.113	<0.001***	632.614	<0.001***	17.534	<0.001***	84.996	<0.001***
Soluble starch	62.166	<0.001***	521.569	<0.001***	39.087	<0.001***	0.827	0.372	2.270	0.145
Total chlorophyll	33.182	<0.001***	363.707	<0.001***	44.717	<0.001***	0.101	0.753	0.383	0.542
Root length	54.824	<0.001***	267.718	<0.001***	87.144	<0.001***	1.431	0.243	0.356	0.556
Root surface area	103.215	<0.001***	333.091	<0.001***	70.327	<0.001***	0.597	0.447	0.010	0.992
Root diameter	163.612	<0.001***	373.405	<0.001***	79.517	<0.001***	1.208	0.283	21.954	<0.001***
Root volume	94.201	<0.001***	259.651	<0.001***	50.556	<0.001***	0.126	0.726	0.438	0.515
Proline	623.135	<0.001***	1338.820	<0.001***	708.966	<0.001***	59.861	<0.001***	151.005	<0.001***
MDA	52.863	<0.001***	159.984	<0.001***	156.637	<0.001***	0.026	0.874	1.969	0.173
SOD	201.193	<0.001***	640.474	<0.001***	232.061	<0.001***	5.522	0.027*	13.702	0.001**
PPO	35.823	<0.001***	349.397	<0.001***	166.498	<0.001***	0.640	0.432	30.807	<0.001***
POD	67.416	<0.001***	111.517	<0.001***	91.877	<0.001***	15.872	0.001**	20.124	<0.001***
CAT	234.239	<0.001***	481.680	<0.001***	9.434	0.005	23.627	<0.001***	0.676	0.419
JA	31.773	<0.001***	656.791	<0.001***	846.362	<0.001***	4.823	0.038*	226.047	<0.001***
SA	68.411	<0.001***	521.844	<0.001***	631.001	<0.001***	23.447	<0.001***	0.019	0.891
Total phenols	53.009	<0.001***	441.461	<0.001***	103.605	<0.001***	0.001	0.993	2.393	0.135
Flavonoids	26.624	<0.001***	117.333	<0.001***	147.262	<0.001***	0.301	0.588	2.913	0.101
Tannic acid	0.089	0.769	0.353	0.558	629.541	<0.001***	0.002	0.998	0.173	0.682

*P < 0.05; **P < 0.01; ***P < 0.001.

### 3.2 Effects of inoculation with AMF and *A. gossypii* herbivory on the nutrient contents of *A. adenophora*

Inoculation with AMF (SC or CE) significantly enhanced the soluble sugar, protein, starch, and total chlorophyll contents of *A. adenophora* without *A. gossypii* infestation (soluble sugar: SC: *F*_(2,24)_ = 35.862, *P* < 0.001, CE: *F*_(2,24)_ = 329.338, *P* < 0.001; soluble protein: SC: *F*_(2,24)_ = 135.392, *P* < 0.001, CE: *F*_(2,24)_ = 373.113, *P* < 0.001; soluble starch: SC: *F*_(2,24)_ = 62.166, *P* < 0.001, CE: *F*_(2,24)_ = 521.569, *P* < 0.001; total chlorophyll: SC: *F*_(2,24)_ = 33.182, *P* < 0.001, CE: *F*_(2,24)_ = 363.707, *P* < 0.001, **Table 1**). Feeding of *A. gossypii* significantly reduced the nutrient contents of *A. adenophora* (soluble sugar: *F*_(2,24)_ = 135.764, *P* = 0.001; soluble protein: *F*_(2,24)_ = 632.614, *P* < 0.001; soluble starch: *F*_(2,24)_ = 39.087, *P* < 0.001; total chlorophyll: *F*_(2,24)_ = 44.717, *P* < 0.001; **Table 1**). However, AM fungal inoculation could increase the nutrient content of *A. adenophora* with *A. gossypii* infestation, and the nutrient content was significantly higher among treatments ranked CE > SC > C (*P* < 0.05, **Table 2**). The effect of CE treatment on the soluble sugar and soluble protein of *A. adenophora* with infestation *A. gossypii* was greater than that of SC treatment (soluble sugar: SC*A. gossypii: *F*_(2,24)_ = 0.014, *P* = 0.907, CE*A. gossypii: *F*_(2,24)_ = 7.893, *P* = 0.010; soluble protein: SC*A. gossypii: *F*_(2,24)_ = 17.534, *P* < 0.001, CE*A. gossypii: *F*_(2,24)_ = 84.996, *P* < 0.001, **Table 1**).

**Table 2:** Effect of inoculation with AMF and *A. gossypii* herbivory on the nutrient composition of *A. adenophora.*.

Inoculation treatment	*A. gossypii* treatments	Soluble sugar (μg/mL)	Soluble protein (μg/mL)	Soluble starch (μg/mL)	Total chlorophyll (mg/g)
C	without *A. gossypii* infestation	9.213 ± 0.248Ac	3.283 ± 0.452Ac	7.231 ± 0.214Ac	3.645 ± 0.205Ac
	with *A. gossypii* infestation	8.179 ± 0.369Bc	1.609 ± 0.229Bc	6.416 ± 0.378Bc	2.935 ± 0.179Bc
SC	without *A. gossypii* infestation	9.999 ± 0.317Ab	5.777 ± 0.125Ab	8.410 ± 0.357Ab	4.541 ± 0.370Ab
	with *A. gossypii* infestation	8.996 ± 0.329Bb	2.783 ± 0.103Bb	7.902 ± 0.238Bb	3.729 ± 0.201Bb
CE	without *A. gossypii* infestation	12.017 ± 0.304Aa	7.780 ± 0.669Aa	11.345 ± 0.448Aa	6.527 ± 0.461Aa
	with *A. gossypii* infestation	10.231 ± 0.194Ba	3.201 ± 0.122Ba	10.020 ± 0.533Ba	5.628 ± 0.419Ba

C, inoculation with sterilized AMF, SC, inoculation with S. constrictum, CE, inoculation with C. etunicatum. Different lowercase letters in the same column indicate significant differences among the four treatments (*P* < 0.05). Different uppercase letters indicate significant differences between the treatments with or without A. gossypii infestation (*P* < 0.05).

### 3.3 Effects of inoculation with AMF and *A. gossypii* herbivory on the root growth parameters

The root growth parameters (root length, root surface, root diameter, and root volume) were significantly increased by two kinds of AM fungal inoculation (Root length: SC: *F*_(2,24)_ = 54.824, *P* < 0.001, CE: *F*_(2,24)_ = 267.718, *P* < 0.001; Root surface area: SC: *F*_(2,24)_ = 103.215, *P* < 0.001, CE: *F*_(2,24)_ = 333.091, *P* < 0.001; Root diameter: SC: *F*_(2,24)_ = 163.612, *P* < 0.001, CE: *F*_(2,24)_ = 373.405, *P* < 0.001; Root volume: SC: *F*_(2,24)_ = 94.201, *P* < 0.001, CE: *F*_(2,24)_ = 259.651, *P* < 0.001, **Table 1**). Feeding of *A. gossypii* significant decreased the root growth parameters of *A. adenophora* with *A. gossypii* infested (Root length: *F*_(2,24)_ = 87.144, *P* < 0.001; Root surface area: *F*_(2,24)_ = 70.327, *P* < 0.001; Root diameter: *F*_(2,24)_ = 79.517, *P* < 0.001; Root volume: *F*_(2,24)_ = 50.556, *P* < 0.001, **Table 1**). AM fungal inoculation could increase the root growth of *A. adenophora* with *A. gossypii* infestation, the root growth parameters with SC treatment increased by 29.86%, 90.99%, 45.91%, 161.37, and CE treatment increased by 75.88%, 177.86%, 117.01% and 289.42%, respectively, compared with uninoculated treatment (*P* < 0.001, **Table 3**). The effect of CE treatment on the root diameter of *A. adenophora* with infestation *A. gossypii* was greater than that of SC treatment (SC*A. gossypii: *F*_(2,24)_ = 1.208, *P* = 0.283, CE*A. gossypii: *F*_(2,24)_ = 21.954, *P* < 0.001, **Table 1**).

**Table 3:** Effect of inoculation with AMF and *A. gossypii* herbivory on the root growth characteristics of *A. adenophora*.

Inoculation treatment	*A. gossypii* treatments	Root length (m)	Root surface area (cm^2^)	Root diameter (mm)	Root volume (cm^3^)
C	without *A. gossypii* infestation	81.179 ± 3.115Ac	524.384 ± 58.501Ac	0.433 ± 0.017Ab	3.052 ± 0.387Ac
	with *A. gossypii* infestation	61.879 ± 5.111Bc	320.241 ± 59.525Bc	0.342 ± 0.041Bc	1.4654 ± 0.419Bc
SC	without *A. gossypii* infestation	106.811 ± 8.642Ab	863.735 ± 59.502Ab	0.775 ± 0.053Aa	5.596 ± 0.670Ab
	with *A. gossypii* infestation	80.349 ± 6.247Bb	611.622 ± 50.318Bb	0.499 ± 0.027Bb	3.829 ± 0.435Bb
CE	without *A. gossypii* infestation	131.721 ± 7.799Aa	1087.864 ± 108.679Aa	0.789 ± 0.053Aa	6.958 ± 0.839Aa
	with *A. gossypii* infestation	108.832 ± 7.489Ba	889.827 ± 63.705Ba	0.740 ± 0.043Ba	5.706 ± 0.495Ba

C, inoculation with sterilized AMF, SC, inoculation with S. constrictum, CE, inoculation with C. etunicatum. Different lowercase letters in the same column indicate significant differences among the four treatments (*P* < 0.05). Different uppercase letters indicate significant differences between the treatments with or without A. gossypii infestation (*P* < 0.05).

### 3.4 Effects of inoculation with AMF and *A. gossypii* herbivory on the proline and MDA contents of *A. adenophora*

**Figure 2:**
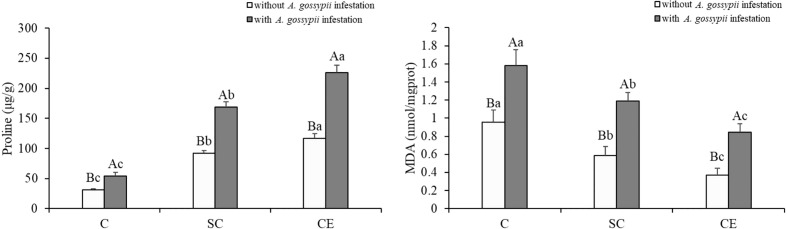
Effect of inoculation with AMF and *A. gossypii* herbivory on the proline and MDA contents of *A. adenophora.* The three treatments were (1) C, inoculation with sterilized AMF, (2) SC, inoculation with *S. constrictum*, (3) CE, inoculation with *C. etunicatum*. Different lowercase letters above the bars indicate significant differences among the three inoculation treatments (Duncan’s test, *P* < 0.05). Different uppercase letters above the bars indicate significant differences between treatments with or without *A. gossypii* infestation (*t*-test, *P* < 0.05) Error bars represent ± 1SE of the mean (n = 5).

Inoculation of two kinds of AMF significantly increased the proline content but decreased the MDA content (Pro: SC: *F*_(2,24)_ = 623.135, *P* < 0.001, CE: *F*_(2,24)_ = 1338.820, *P* < 0.001; MDA: SC: *F*_(2,24)_ = 52.863, *P* < 0.001, CE: *F*_(2,24)_ = 159.984, *P* < 0.001, **Table 1**). Feeding of *A. gossypii* significantly increased proline and MDA contents of *A. adenophora* (Pro: *F*_(1,24)_ = 708.966, *P* < 0.001; MDA: *F*_(1,24)_ = 156.637, *P* < 0.001, **Table 1**), the proline content increased by 63.84% and the MDA content increased by 64.93% (**Figure 2**). Compared with non-inoculated treatment with *A. gossypii* infestation, the proline content of *A. adenophora* inoculated with SC and CE increased by 210.60% and 314.88% (*P* < 0.001, **Figure 2**), while the MDA content in the SC and CE treatment decreased by 24.62% and 46.58% (*P* < 0.001, **Figure 2**), respectively. The effect of CE treatment on the proline content of *A. adenophora* with infestation *A. gossypii* was greater than that of SC treatment (SC*A. gossypii: *F*_(2,24)_ = 59.861, *P* < 0.001, CE*A. gossypii: *F*_(2,24)_ =151.005, *P* < 0.001, **Table 1**).

### 3.5 Effects of inoculation with AMF and *A. gossypii* herbivory on the antioxidant enzyme activities of *A. adenophora*

**Figure 3:**
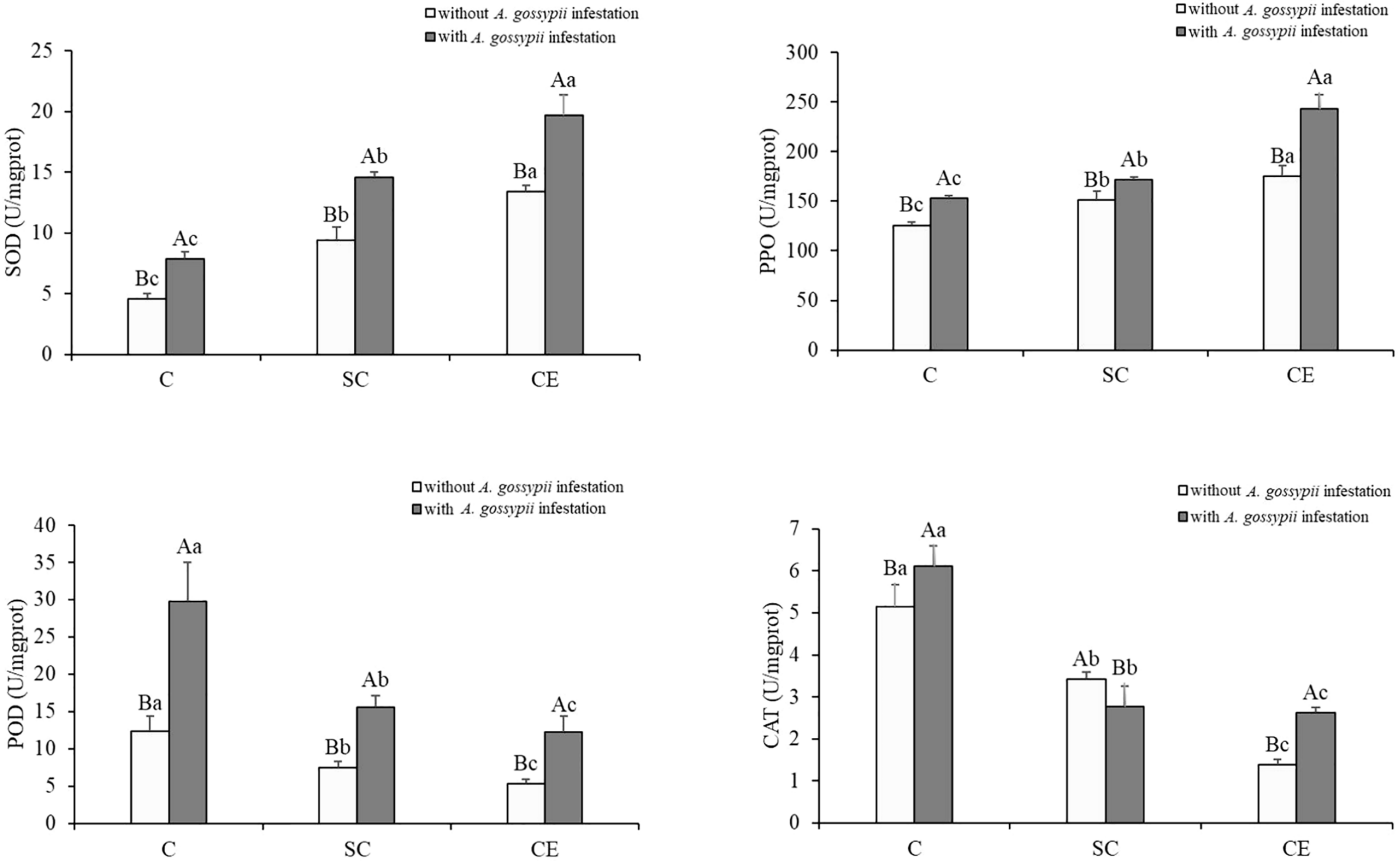
Effect of inoculation with AMF and *A*. *gossypii* herbivory on the antioxidant enzyme activity (SOD, PPO, POD and CAT) in *A. adenophora.* The three treatments were (1) C, inoculation with sterilized AMF, (2) SC, inoculation with *S. constrictum*, (3) CE, inoculation with *C. etunicatum*. Different lowercase letters above the bars indicate significant differences among the three inoculation treatments (Duncan’s test, *P* < 0.05). Different uppercase letters above the bars indicate significant differences between treatments with or without *A. gossypii* infestation (*t*-test, *P* < 0.05) Error bars represent ± 1SE of the mean (n = 5).

The activities of SOD and PPO were significantly increased, but the activities of POD and CAT were significantly decreased by two kinds of AM fungal inoculation (SOD: SC: *F*_(2,24)_ = 201.193, *P* < 0.001, CE: *F*_(2,24)_ = 640.474, *P* < 0.001; PPO: SC: *F*_(2,24)_ = 35.823, *P* < 0.001, CE: *F*_(2,24)_ = 349.397, *P* < 0.001; POD: SC: *F*_(2,24)_ = 67.416, *P* < 0.001, CE: *F*_(2,24)_ = 111.517, *P* < 0.001; CAT: SC: *F*_(2,24)_ = 234.239, *P* < 0.001, CE: *F*_(2,24)_ = 481.680, *P* < 0.001, **Table 1**). Feeding of *A. gossypii* significantly increased the activities of all antioxidant enzyme in *A. adenophora* (SOD: *F*_(1,24)_ = 232.061, *P* < 0.001; PPO: *F*_(1,24)_ = 166.498, *P* < 0.001; POD: *F*_(1,24)_ = 91.877, *P* < 0.001; CAT: *F*_(1,24)_ = 9.434, *P* = 0.005, **Table 1**). The activities of SOD, PPO, POD, and CAT were increased by 72.18%, 21.27%, 140.02%, and 18.78% in the *A. gossypii* infestation treatment compared with the treatment without *A. gossypii* infestation. With *A. gossypii* infestation, the SOD activity in *A. adenophora* in the SC and CE inoculation treatments increased by 86.93% and 148.53% compared with the uninoculated treatment, and the PPO activity increased by 12.62% and 57.85% (*P* < 0.05, **Figure 3**), respectively. The POD activity in *A. adenophora* in the SC and CE inoculation treatments decreased by 46.31% and 57.52% compared with the uninoculated treatment with *A. gossypii* infestation, and the CAT activity decreased by 42.70% and 57.07% (*P* < 0.05, **Figure 3**), respectively. The effect of CE treatment on the SOD, PPO, POD activity of *A. adenophora* with infestation *A. gossypii* was greater than that of SC treatment (SOD: SC*A. gossypii: *F*_(2,24)_ = 5.522, *P* = 0.027, CE*A. gossypii: *F*_(2,24)_ =13.702, *P* < 0.001; PPO: SC*A. gossypii: *F*_(2,24)_ = 0.640, *P* = 0.432, CE*A. gossypii: *F*_(2,24)_ =30.807, *P* < 0.001; POD: SC*A. gossypii: *F*_(2,24)_ = 15.872, *P* = 0.001, CE*A. gossypii: *F*_(2,24)_ =20.124, *P* < 0.001, **Table 1**).

### 3.6 Effects of inoculation with AMF and *A. gossypii* herbivory on the defense hormones contents of *A. adenophora*

**Figure 4:**
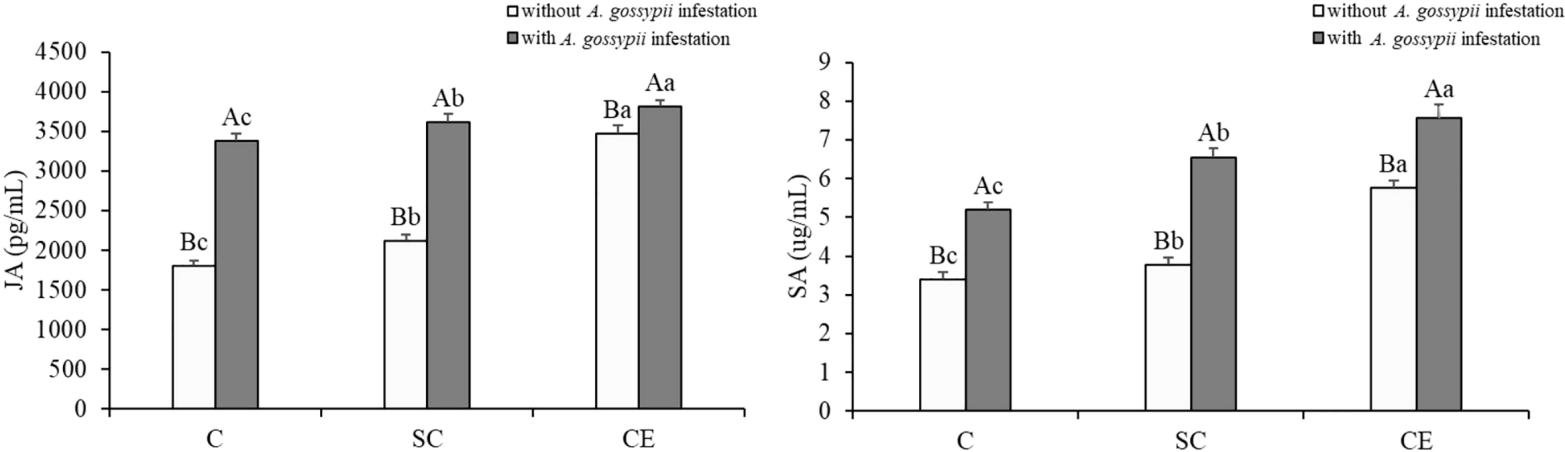
Effect of inoculation with AMF and *A*. *gossypii* herbivory on the jasmonic acid (JA) and salicylic acid (SA) contents of *A. adenophora.* The three treatments were (1) C, inoculation with sterilized AMF, (2) SC, inoculation with *S. constrictum*, (3) CE, inoculation with *C. etunicatum*. Different lowercase letters above the bars indicate significant differences among the three inoculation treatments (Duncan’s test, *P* < 0.05). Different uppercase letters above the bars indicate significant differences between treatments with or without *A. gossypii* infestation (*t*-test, *P* < 0.05) Error bars represent ± 1SE of the mean (n = 5).

The defensive hormones contents of *A. adenophora* with the two kinds of AM fungal treatments were significantly increased (JA: SC: *F*_(2,24)_ = 31.773, *P* < 0.001, CE: *F*_(2,24)_ = 656.791, *P* < 0.001; SA: SC: *F*_(2,24)_ = 68.411, *P* < 0.001, CE: *F*_(2,24)_ = 521.844, *P* < 0.001, **Table 1**). Feeding of *A. gossypii* significantly increased the JA and SA contents of *A. adenophora* (JA: *F*_(1,24)_ = 846.352, *P* < 0.001; SA: *F*_(1,24)_ = 631.001, *P* = 0.001, **Table 1**). The JA and SA content increased by 87.62% and 52.58% in the *A. gossypii* infestation treatment (**Figure 4**). The JA contents in the SC- and CE-inoculated treatments increased by 4.92% and 13.49%, and the SA contents in the SC- and CE-inoculated treatments increased by 25.07% and 43.29% with *A. gossypii* infestation (*P* < 0.001, **Figure 4**), respectively. The effect of CE treatment on the JA of *A. adenophora* with infestation *A. gossypii* was greater than that of SC treatment (SC*A. gossypii: *F*_(2,24)_ = 4.823, *P* = 0.038, CE*A. gossypii: *F*_(2,24)_ =226.047, *P* < 0.001, **Table 1**).

### 3.7 Effects of inoculation with AMF and *A. gossypii* herbivory on the secondary metabolite contents of *A. adenophora*

**Figure 5:**
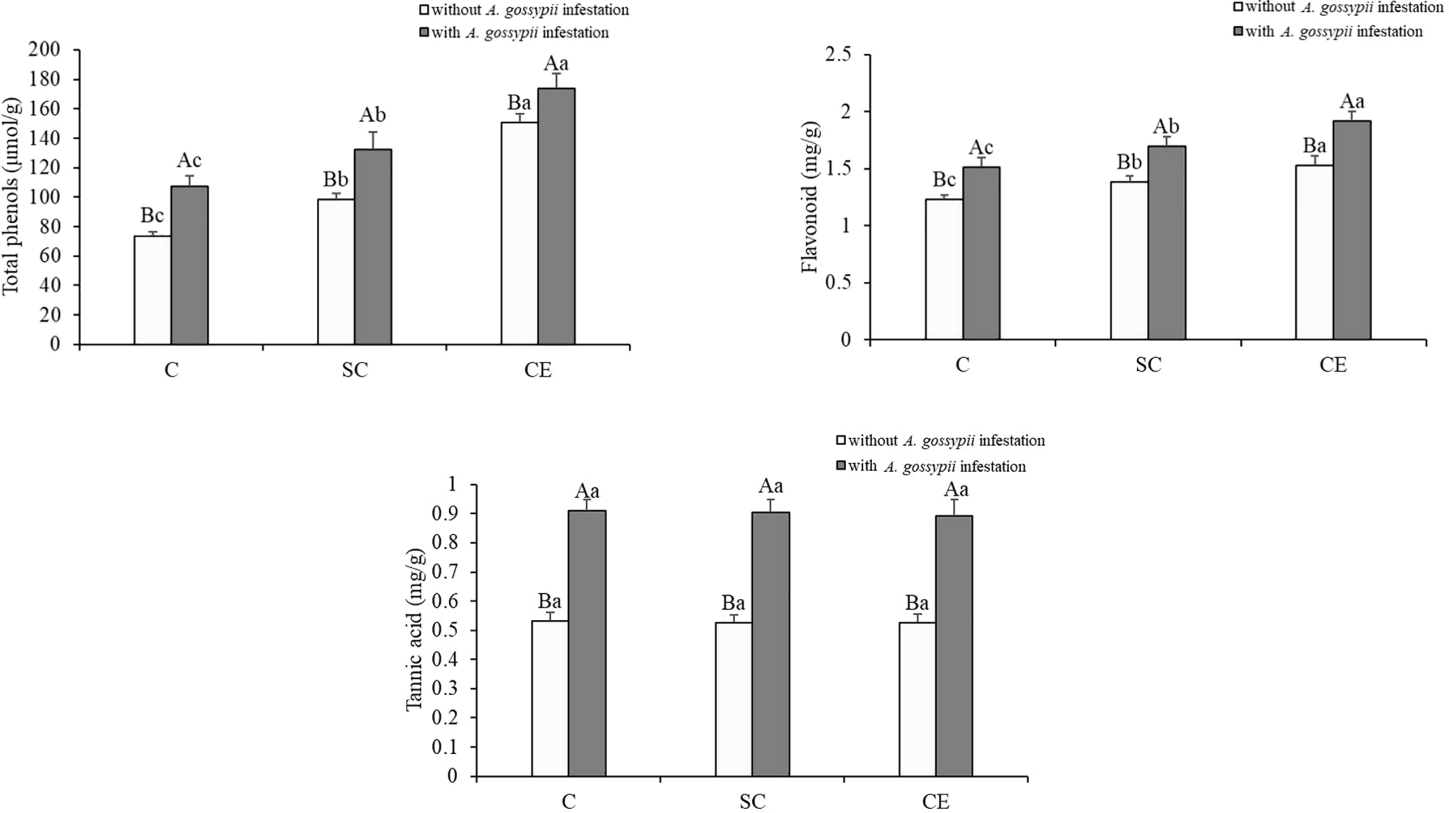
Effect of inoculation with AMF and *A*. *gossypii* herbivory on the secondary metabolite (total phenols, flavonoid and tannic acid) contents of *A. adenophora*. The three treatments were (1) C, inoculation with sterilized AMF, (2) SC, inoculation with *S. constrictum*, (3) CE, inoculation with *C. etunicatum*. Different lowercase letters above the bars indicate significant differences among the three inoculation treatments (Duncan’s test, *P* < 0.05). Different uppercase letters above the bars indicate significant differences between treatments with or without *A. gossypii* infestation (*t*-test, *P* < 0.05) Error bars represent ± 1SE of the mean (n = 5).

Inoculation with two kinds of AMF significantly increased the total phenolic and flavonoids contents of *A. adenophora* (total phenols: SC: *F*_(2,24)_ = 53.009, *P* < 0.001, CE: *F*_(2,24)_ = 441.461, *P* < 0.001; flavonoids: SC: *F*_(2,24)_ = 26.624, *P* < 0.001, CE: *F*_(2,24)_ = 117.333, *P* < 0.001) but had no significant effect on the tannic acid content (**Table 1**). Feeding of *A. gossypii* significantly increased all secondary metabolite contents of *A. adenophora* (total phenols: *F*_(1,24)_ = 117.332, *P* < 0.001; flavonoids: *F*_(1,24)_ = 149.888, *P* = 0.001; tannic acid: *F*_(1,24)_ = 683.468, *P* < 0.001, **Table 1**). The content of total phenols, flavonoids and tannic acid were increased by 45.66%, 34.09%, and 15.36% in the *A. gossypii* infestation treatment compared with the treatment without *A. gossypii* infestation (**Figure 5**). With *A. gossypii* infestation, the total phenols content of *A. adenophora* in the SC- and CE-inoculated treatments increased by 46.31% and 57.52% compared with the uninoculated treatment (**Figure 5**), and the flavonoid content increased by 11.92% and 27.79% (*P* < 0.05, **Figure 5**), respectively.

### 3.8 Effects of different AMF and *A. gossypii* herbivory on the colonization rate of AMF in the roots of *A. adenophora*

**Figure 6:**
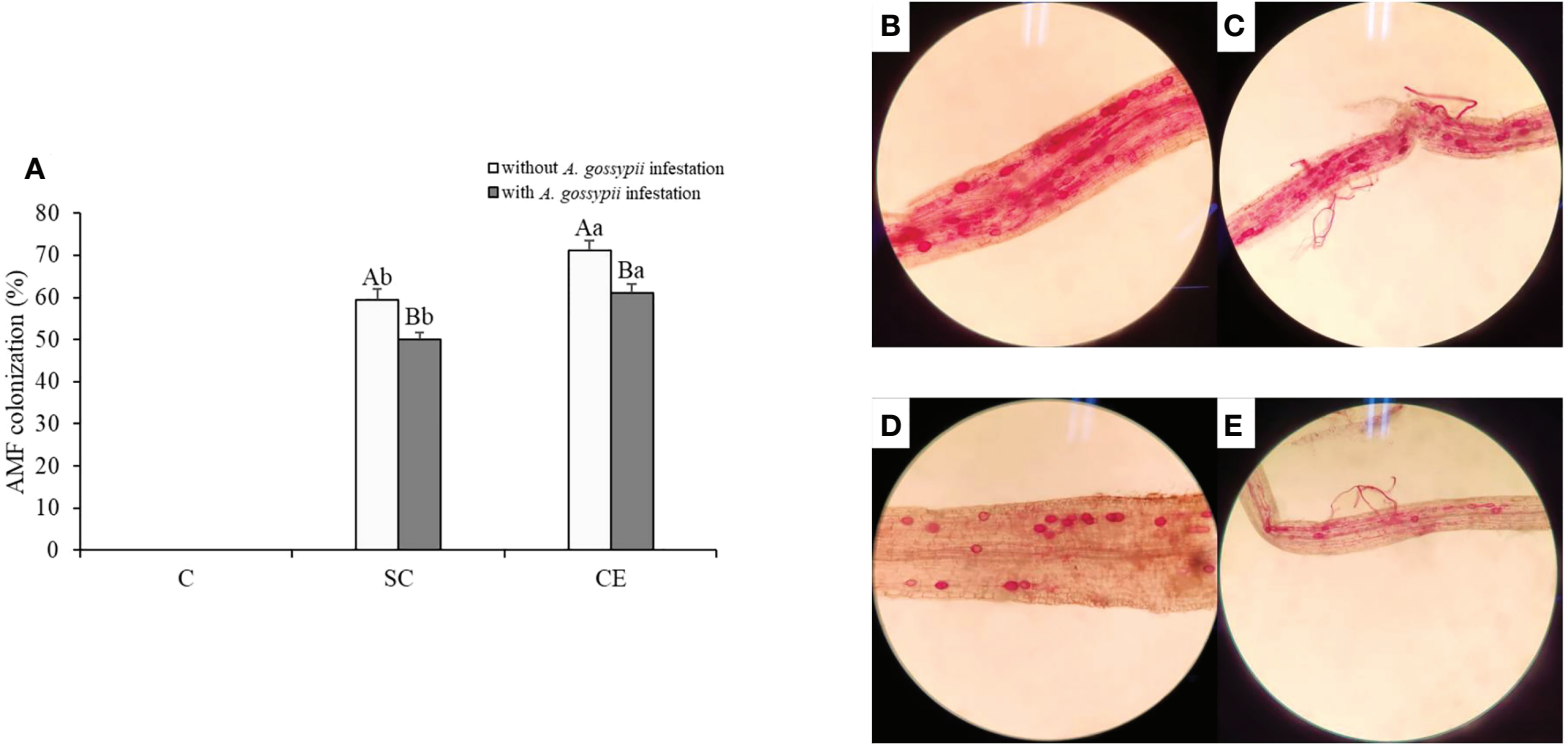
**(A)** Effect of inoculation with AMF and *A. gossypii* herbivory on the AM colonization of *A. adenophora.* The three treatments were (1) C, inoculation with sterilized AMF, (2) SC, inoculation with *S. constrictum*, (3) CE, inoculation with *C. etunicatum*. Different lowercase letters above the bars indicate significant differences among the three inoculation treatments (one-way ANOVA followed by Duncan’s test, *P* < 0.05). Different uppercase letters above the bars indicate significant differences between treatments with or without *A. gossypii* infestation (*t*-test, *P* < 0.05) Error bars represent ± 1SE of the mean (n = 5). **(B–E)**. Representative light micrographs (images) of colonization patterns by indigenous arbuscular mycorrhizal fungi in acid fuchsin stained roots of: **(B)** *A. adenophora* in *C. etunicatum* inoculated treatment without *A. gossypii* infestation; **(C)** *A. adenophora* in *C. etunicatum* inoculated treatment with *A. gossypii* infestation; **(D)** *A. adenophora* in *S. constrictum* inoculated treatment without *A. gossypii* infestation; **(E)** *A. adenophora* in *S. constrictum* inoculated treatment with *A. gossypii* infestation.

No AM fungal colonization, mycelium fragments, or AM spores were found in the non-inoculation treatment both with and without *A. gossypii* treatments (**Figure 6**). The colonization of SC and CE treatments was 59.38% and 71.25% without *A. gossypii* infestation and were 49.95% and 61.12% with *A. gossypii* infestation (**Figure 6**), respectively. The results showed that *A. gossypii* infestation significantly reduced the AM fungal colonization of *A. adenophora* (*P* < 0.001), and different AMF had different colonization levels in *A. adenophora*.

### 3.9 Effects of different AM fungal inoculation on the *A. gossypii* survival rate and fecundity

**Figure 7:**
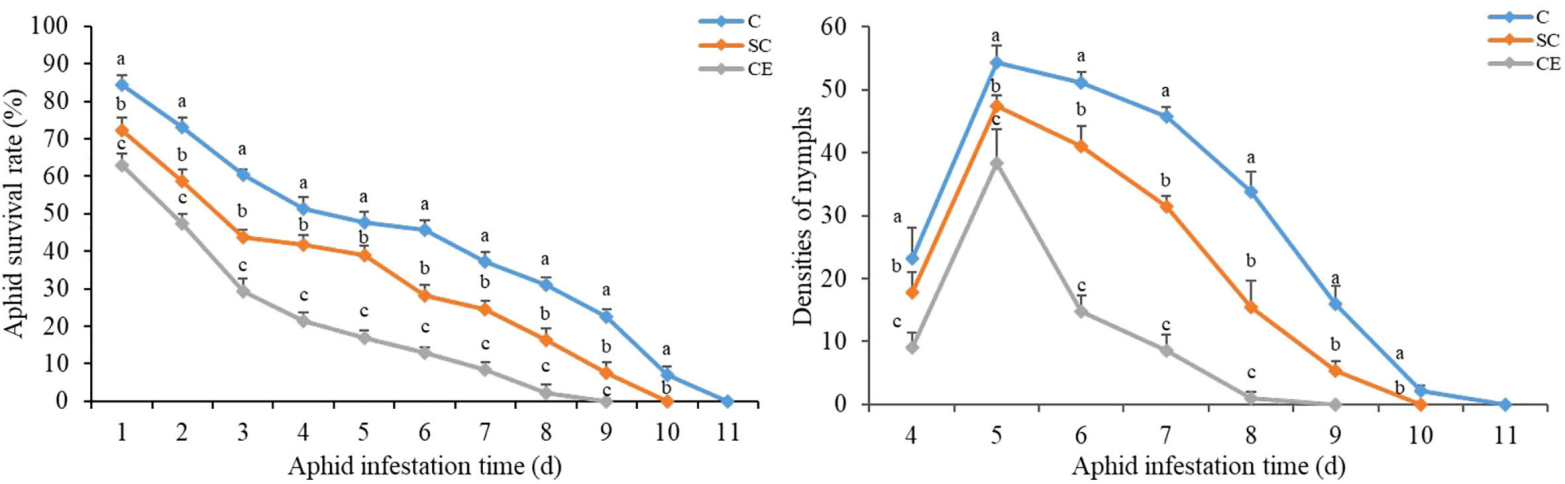
Effect of different AMF inoculation on the *A. gossypii* survival rate and the densities of nymphs. The three treatments were (1) C, inoculation with sterilized AMF, (2) SC, inoculation with *S. constrictum*, (3) CE, inoculation with *C. etunicatum*. Different lowercase letters above the bars indicate significant differences among the three inoculation treatments (one-way ANOVA followed by Duncan’s test, *P* < 0.05). Values are means and bars indicate SE.

On the first day after inoculation of *A. gossypii*, many aphids had died in SC and CE treatment, and the survival rates were 72.33% and 63%, respectively, and the survival rate of non-inoculation treatment was 84.33% (**Figure 7**). The *A. gossypii* survival rate both in the AM fungal inoculation treatment was significantly lower than that in the non-inoculation treatment (*P* < 0.001). All the aphids on the plants inoculated with CE were dead on the 9^th^ day, and the aphids that of inoculated with SC were dead on the 10^th^ day, while the aphids that of non-inoculated were dead on the 11^th^ day (**Figure 7**). For the densities of nymphs, the production of nymphs was found in both three inoculation treatments on the 4^th^ day (**Figure 7**). On the 5th day, the densities of nymphs increased significantly in all three treatments, but the densities of aphid production in the SC and CE treatment was significantly lower than that in the non-inoculated treatment (*P* < 0.001). On the 9^th^ day, no nymphs were found in CE inoculated treatment and aphid reproduction stopped, aphid reproduction stopped on the 10^th^ day and 11^th^ day in SC inoculated treatment and non-inoculated treatment, respectively (**Figure 7**). The results showed that inoculation with AMF had an inhibitory effect on the growth of *A. gossypii* on *A. adenophora*.

## Discussion

Our study investigated the effect of the dominant AMF in the roots of *A. adenophora* on the response of this species to *A. gossypii* feeding. Our results showed that the *A. gossypii* feeding affects the growth of *A. adenophora* and resulted in the plant response to stress. However, the inoculation of two kinds of AMF (*S. constrictum* and *C. etunicatum*) not only significantly promoted the growth of *A. adenophora* (**Figure 1**; **Tables 2**, **3**) but also induced plant defenses, resulting in quicker or stronger stress responses to herbivores (**Figures 2**–**5**). Plants with different AM fungal species differed in their resistance to herbivores depending on the species of AMF with which they were associated (Delavaux et al., 2017; Frew and Wilson, 2021). Bennett et al. (2009) found that AM fungal species’ identity and composition can strongly affect the plant defensive phenotype. We found that the effects of inoculation with *C. etunicatum* on the growth and defense against insect herbivory of *A. adenophora* were significantly higher than those of inoculation with *S. constrictum*, and the colonization of *C. etunicatum* was significantly higher than that of *S. constrictum* (**Figure 6**), indicating that *C. etunicatum* had stronger adaptability for herbivory resistance of *A. adenophora*.

The plant-AM fungal interaction improves plant nutrition, thereby increasing biomass and enhancing the tolerance of the plant to herbivory (Heijden et al., 2015; Sharma et al., 2017). In our study, inoculation with both two AMF increased the biomass, soluble sugar, soluble protein, soluble starch, and chlorophyll contents of *A. adenophora* (**Figure 1** and **Table 2**). Hempel et al. (2009) found that inoculation with AMF (*Glomus intraradices* and *G. mosseae*) generally increased plant biomass and reduced aphid population growth. Our results showed that *A. gossypii* feeding decreased root growth of *A. adenophora*, while the root biomass, root surface area, and root diameter were significantly increased when inoculated with AM fungi (**Figure 1** and **Table 3**). AMF can directly promote root growth which could increase the absorption and delivery of nutrients, especially nitrogen and phosphorus (Wang et al., 2017b; Wipf et al., 2019). Tao et al. (2016) showed that the tolerance of different milkweed plants to herbivory increased with the foliar P concentration when colonized by AMF. Insect herbivory reduces plant biomass, damages leaves, and reduces photosynthesis, leading to plant wilting and death (Chen et al., 2017). The chlorophyll content is higher in mycorrhizal plants, which is mainly due to the higher nitrogen content of the plants, and the high nutrient content can improve the stress resistance of the plants (Mathur et al., 2018). Odebode and Salami (2004) reported that increased levels of sugars and free amino acids in pepper seedlings inoculated with AMF increased plant defense.

The oxidative shift is indicated by increasing levels of oxidative enzymes, reactive oxygen species, and proline content, as well as decreasing MDA and nutritional antioxidants such as ascorbate (Vannini et al., 2016). Plants defend themselves through oxidative shifts to produce oxidative and nutritional stress in herbivores (Cao et al., 2016). We found that inoculation with AMF increased the proline content of *A. adenophora* and decreased the MDA content, thus maintaining the normal growth of the *A. adenophora* in response to the damage on *A. gossypii* feeding (**Figure 2**). Sun et al. (2020) also found that when *Procecidochares utilis* parasitizes *A. adenophora*, the MDA content of *A. adenophora* decreases and the proline content increases, which increases the free radical scavenging activity. Such changes in the activities of oxidative enzymes in the plant cause a decrease in herbivory and provide induced resistance to the plant in response to pest infestation (Zhang et al., 2008). Our study also found that inoculation with AMF significantly increased the activity levels of SOD and PPO of *A. adenophora* (**Figure 3**). SOD of plants can exert its unique effect to remove a serious of reactive oxide species (ROS) caused by insect herbivory to maintain plant’s ordinary (Khan et al., 2021). PPO is thought to be an effective defense enzyme against insects (War et al., 2012). Higher activity of PPO could lead to a higher production of quinones and the formation of lignin, which affects the synthesis of cell wall (Kaur et al., 2015). Balog et al. (2017) found that AMF can enhance PPO activity in peppers and decrease infestation by arthropod pests. Thus, we infer that AMF may be able to defend against herbivory of *A. gossypii* by increasing PPO activity in *A. adenophora* to produce quinones and lignin from phenolics through oxidation.

Through interaction with phytophagous insects, plants have evolved defense mechanisms to reduce herbivore damage, and the induced resistance has broad-spectrum and rapid action, which can enhance plant defense against insect attacks (Kawazu et al., 2013; Mauch-Mani et al., 2017). Several studies have revealed that plant hormones mediate the induced resistance of plants to biological attack, among which jasmonic acid and salicylic acid are the most critical (Pozo et al., 2015; Kadam et al., 2020). Our study found that AM fungal inoculation significantly increased the contents of JA and SA that prime plant-inducible defense (**Figure 4**). Jiang et al. (2021) showed that three JA biosynthesis-related genes (lipoxygenase LOX, hydroperoxide dehydratase AOS, allene oxide cyclase AOC) and two JA signal transduction-related genes (jasmonate-ZIM domain JAZ, transcription factor MYC2) were upregulated in mycorrhizal *P. alba ×P. berolinensis* seedlings compared with nonmycorrhizal seedlings, resulting in a high JA content in plant tissues. The increase in the JA content induces chemical defense in plants, regulates the expression of plant secondary metabolite genes, and promotes the synthesis and accumulation of secondary metabolites, thus reducing herbivore preference, performance and abundance (Zhang et al., 2022). We found that the content of total phenols and flavonoids in *A. adenophora* was significantly increased after inoculation with AMF (**Figure 5**). Several studies have demonstrated that AMF inoculation increase the insecticidal metabolites in plants, with phenolic compounds being the most abundant class of all identified metabolites, including flavonoids and tannins. These substances can directly affect the growth, survival and reproduction of feeding insects (Züst and Agrawal, 2017; Wang et al., 2019; Frew et al., 2022). Our results showed that the survival and reproduction of *A. gossypii* on *A. adenophora* inoculated with AM fungi were inhibited (**Figure 7**), which may be due to the increased content of total phenolic and flavonoid substances in the leaves, and thus causing toxic effect on *A. gossypii*. AMF can drive insect herbivore performance by affecting phenolic-based resistance mechanisms, which are mainly manifested by increasing foliar phenolic compounds and reducing the relative growth rate of herbivorous insects (Jiang et al., 2021; Frew and Wilson, 2021).

In summary, the symbiosis of the two dominant AMF in the rhizosphere of *A. adenophora* not only improved the tolerance of *A. adenophora* by increasing the biomass and nutrient contents, but also maintain the plant’s ordinary by increasing the proline content and SOD activity, and inhibited the feeding of *A. gossypii* by increasing the PPO activity. It can also induce the production and accumulation of secondary metabolites (jasmonic acid and salicylic acid), which reduced the survival rate and reproductive ability of *A. gpssypii*. Moreover, the effect on insect resistance of *A. adenophora* by *C. etunicatum* inoculation higher than that by *S. constrictum*. In the invasion process of *A. adenophora*, the community structure of AMF could change to improve its competitive ability and defense against generalist herbivores in the introduced ranges, and the AM fungal colonization rate of native plants and resistance to insects could be reduced, leading to the rapid expansion and outbreak of *A. adenophora* in the introduced area (Inderjit, 2012). This laid a foundation for us to enhance the biological control effect of *A*. *gossypii* on *A. adenophora* by controlling the abundance of dominant AMF in the rhizosphere or changing the community structure of AMF in the rhizosphere of *A. adenophora*.

## Data availability statement

The original contributions presented in the study are included in the article/supplementary material. Further inquiries can be directed to the corresponding author.

## Author contributions

FG, ED, and YC designed the research. FZ and RH collected the samples. ED and YL performed the experiments. ZS and YL performed bioinformatic and statistical analyses. ED and YC wrote the first draft. ZS and FG reviewed the manuscript. All authors contributed to the article and approved the submitted version.

## Funding

This work was supported by the Yunnan Eco-Friendly Food International Cooperation Research Center (YEFICRC) Project of Yunnan Provincial of Yunnan Provincial Key Program (Grant No.2019ZG00910), National Key Research and Development Program of China (2021YFD1400200), National Nature Science Foundation of China (NSFC) (31660546), and Scientific Research Foundation of Education Department of Yunnan Province (2022Y224).

## Conflict of interest

The authors declare that the research was conducted in the absence of any commercial or financial relationships that could be construed as a potential conflict of interest.

## Publisher’s note

All claims expressed in this article are solely those of the authors and do not necessarily represent those of their affiliated organizations, or those of the publisher, the editors and the reviewers. Any product that may be evaluated in this article, or claim that may be made by its manufacturer, is not guaranteed or endorsed by the publisher.
